# Ursolic acid induces the production of IL6 and chemokines in both adipocytes and adipose tissue

**DOI:** 10.1080/21623945.2020.1814545

**Published:** 2020-09-02

**Authors:** Bin Feng, Yingguo Zhu, Lijun Yan, Hui Yan, Xiaohua Huang, Dandan Jiang, Zhen Li, Lun Hua, Yong Zhuo, Zhengfeng Fang, Lianqiang Che, Yan Lin, Shengyu Xu, Chao Huang, Yuanfeng Zou, Lixia Li, De Wu

**Affiliations:** a Animal Nutrition Institute, Sichuan Agricultural University, Chengdu, Sichuan, China; b Key Laboratory of Animal Disease-Resistant Nutrition of Ministry of Education, Sichuan Agricultural University, Chengdu, Sichuan, China; c Key Laboratory of Animal Disease-Resistant Nutrition of Sichuan Province, Sichuan Agricultural University, Chengdu, Sichuan, China; d College of Veterinary Medicine, Sichuan Agricultural University, Chengdu, Sichuan, China

**Keywords:** Ursolic acid, adipocyte, IL6, chemokine, MCP1, monocyte, TLR4

## Abstract

Adipose tissue inflammation plays an important role in the regulation of glucose and lipids metabolism. It is unknown whether Ursolic acid (UA) could regulate adipose tissue inflammation, though it can regulate inflammation in many other tissues. In this study, 3T3-L1 adipocytes, DIO mice and lean mice were treated with UA or vehicle. Gene expression of inflammatory factors, chemokines and immune markers in adipocytes and adipose tissue, cytokines in cell culture medium and serum, and inflammation regulatory pathways in adipocytes were detected. Results showed that UA increased the expression of interleukins and chemokines, but not TNFα, in both adipocytes and adipose tissue. IL6 and MCP1 levels in the cell culture medium and mouse serum were induced by UA treatment. Cd14 expression level and number of CD14+ monocytes were higher in UA treated adipose tissue than those in the control group. Glucose tolerance test was impaired by UA treatment in DIO mice. Mechanistically, UA induced the expression of Tlr4 and the phosphorylation levels of ERK and NFκB in adipocytes. In conclusion, our study indicated that short-term UA administration could induce CD14+ monocytes infiltration by increasing the production of interleukins and chemokines in mouse adipose tissue, which might further impair glucose tolerance test.

## Introduction

Adipose tissue plays an important role in metabolic health, for it serves as not only a storage organ for excess energy in the form of triacylglycerol but also an endocrine organ for many cytokines, such as interleukin 6 (IL6), tumour necrosis factor α (TNFα), monocyte chemokine protein 1 (MCP1), macrophage inflammatory protein 2 (MIP2), adiponectin and leptin, which regulate systemic insulin sensitivity and energy homoeostasis [1–4]. Lipids homoeostasis in the adipose tissue is mediated by lipogenesis and lipolysis. Lipogenesis, which means lipids synthesis, is mediated by lipogenic enzymes, such as fatty acid synthetase (FASN), stearoyl-CoA desaturase 1 (SCD1) and acetyl CoA carboxylase (ACC). Lipolysis, which means fat mobilization, is mediated by adipose triglyceride lipase (ATGL) and hormone-sensitive lipase (HSL) [5]. Inflammatory factors and chemokines can regulate lipids metabolism in adipose tissue [3,6]. IL6 and TNFα induce lipolysis and suppress lipogenesis, while chemokines mediate adipose tissue to recruit macrophages and monocytes, which can produce more inflammatory factors to impair adipose homoeostasis. Dysfunction of adipose tissue, especially adipose tissue inflammation, contribute to the development of insulin resistance and metabolic disorders [6–8].

Ursolic acid (UA), a pentacyclic triterpenic acid distributed in a variety of traditional medicine herbs and edible plants, has multiple biological functions, including regulation of glucose and lipids metabolism, anti-cancer, anti-oxidation and anti-inflammation [9]. UA has been proved to ameliorate hepatic steatosis, suppress adipogenesis of pre-adipocytes, and stimulate lipolysis in cultured adipocytes [10–13]. Besides, UA was also reported to regulate inflammation in macrophages, mammary epithelial cells, liver, skin, lung, kidney and intestine, with both anti- and pro-inflammatory effects in different cell types and tissues [9,14–18]. However, the effect of UA on adipose tissue inflammation has not been reported.

Nuclear factor kappa B (NFκB) signalling pathway is a key regulator for the secretion of IL6, TNFα, interleukin 1β (IL1β) and other pro-inflammatory cytokines under the treatment of UA [19,20]. Furthermore, it is reported that the effect of UA on IL6 production was also mediated by toll-like receptor 4 (TLR4) and inhibitor of kappa B kinase beta (IKKβ) in macrophages, by extracellular regulated protein kinases (ERK) in liver, and by Caspase-3 in BEAS-2B cells [21–23].

To clarify the effect of UA on adipose tissue inflammation, we treated both 3T3-L1 adipocytes and mice with UA, and found that short-term UA administration could induce the expression of interleukins, chemokines and inflammation regulatory genes in both cultured adipocytes and adipose tissue. Moreover, UA treatment stimulated the adipose tissue to recruit CD14 positive monocytes.

## Material and methods

### Animal study

Animal study protocol (SICAU-2017-028) was reviewed and approved by the Animal Care and Use Committee of Sichuan Agricultural University. All animal procedures were performed according to the guide for the care and use of laboratory animals of the National Institute of Health. C57BL/6 J male and female mice were obtained from Laboratory Animal Research Centre of Sichuan University (Chengdu, China), and were housed under the temperature of 22 ± 2°C and humidity of 60 ± 5% with 12 h light/dark cycle.

Fifteen 5-week-old female mice with similar body weight were fed with high fat diet (HFD, 60% energy was from fat, Medicience Ltd, Yangzhou, China). At 19-week of age, mice were randomized into 3 groups with similar body weight (BW). Mice in group 1 (Control, n = 5) were intraperitoneally (i.p.) injected with the vehicle (dimethyl sulphoxide, DMSO, Sigma, Shanghai, China), mice in group 2 (UA10, n = 5) were injected with UA (Sigma, Shanghai, China) at 10 mg/kg BW and mice in group 3 (UA20, n = 5) were injected with UA at 20 mg/kg BW.

Thirteen 12-week-old male lean mice with the similar weight were randomly divided into two groups. One group (n = 7) was injected with DMSO at 0.35 mL/kg BW, and the other group (n = 6) was injected with UA at 20 mg/kg BW. All mice were fed with normal chow diet (Dashuo, Chengdu, China).

All mice were injected once daily for 3 d. In the evening of the third day, mice were starved for 12 h. The next morning, mice were sacrificed 2 h after UA or vehicle injection. Bodyweight was recorded, tail vein blood glucose levels were measured with glucose strips (Beijingyicheng, Beijing, China). Blood, gonadal adipose tissues, perirenal adipose tissues and subcutaneous adipose tissues were collected, weighed and frozen in liquid nitrogen, followed by storing at −80°C for further analysis. One piece of gonadal fat tissue was fixed in 4% formalin for haematoxylin and eosin stain and immunohistology stain. The weights of gonadal adipose tissues, perirenal adipose tissues and subcutaneous adipose tissues were summed as weight of total white adipose tissue (WAT).

### GTT and ITT study

Glucose tolerance test (GTT) and insulin tolerance test (ITT) were performed as previously reported [24]. Briefly, 19-week-old female diet-induced obese mice were intraperitoneally injected with UA or vehicle (n = 7 per group) daily. On day 4, mice were intraperitoneally injected with 0.75 g/kg BW glucose (Sigma) after an overnight (12 h) fast. Blood glucose levels were measured with tail-vein blood with glucose strips at 0, 15, 30, 45, 60 and 90 min post glucose injection. Mice were then put back to recover for 3 d with daily injection of UA or vehicle.

ITT was performed on day 7 of UA treatment after 6 h-fasting. Mice were intraperitoneally injected with 1.2 U/kg BW of insulin. Blood glucose levels were measured at 0, 15, 30, 45, 60 and 90 min post insulin injection.

### Cell culture

The culture and differentiation of 3T3-L1 preadipocytes (ATCC) were performed as previously reported [24]. Briefly, 3T3-L1 preadipocytes were cultured in 10% foetal bovine serum (FBS) (Gibco, Shanghai, China), 100 U/mL penicillin and 100 μg/mL streptomycin (Gibco, Shanghai, China) supplemented DMEM (Gibco, Shanghai, China), under the condition of 37°C and 5% CO_2_. Cells were subcultured into a 12-well plate at the density of 5 × 10^5^ per well. Two days after 100% confluence, cells were incubated in 10% FBS containing DMEM, plus 1 µg/mL insulin (Sigma), 1 µM dexamethasone (Sigma) and 0.5 mM isobutyl methylxanthine (Sigma) for 4 d. Cells were then continually maintained in 10% FBS containing DMEM with 1 µg/mL insulin for another 6 d. Medium was freshly changed every other day. Cells were then treated with UA (Sigma) or vehicle (DMSO) after 12 h-incubation in serum-free DMEM.

### Measure of inflammatory cytokines

Serum levels of IL6, TNFα and MCP1 were measured with the respective ELISA kits (BioLegend, San Diego, CA, USA) according to the instructions of manufacturer, as previously reported [24].

For the detection of IL6 and MCP1 levels in the cell culture medium, fully differentiated 3T3-L1 adipocytes were treated with 25 µM UA or vehicle for 12 h in serum-free DMEM. Levels of IL6 and MCP1 in the medium were quantified with the respective ELISA kits (BioLegend).

### Histochemistry and immunohistochemistry straining

The formalin fixed gonadal adipose tissues were embedded in paraffin and were sliced into 4 µm sections. H&E staining was performed as previously reported [24]. Briefly, sections were dehydrated and stained with haematoxylin for 5 min, followed by 2 min staining with eosin after washing with double distilled water. Sections were then dehydrated and mounted onto slides with a neutral resin.

Immunohistochemistry straining was performed by Servicebio (Wuhan, China) using the DAB-stain method. Anti-CD14 antibody (Servicebio, Wuhan, China) was used at the dilution of 1:200.

Images were captured on a microscope (TS100, Nikon, Tokyo, Japan) with a CCD (DS-U3, Nikon, Tokyo, Japan) using imaging software (NIS-Elements F3.2, Nikon).

### RNA extraction and real-time PCR

Total RNA was isolated from gonadal adipose tissues and adipocytes with TRI Reagent (Sigma, Shanghai, China) under the manufacturer’s instruction. The quality of total RNA was assessed by agarose gel and the concentration was measured with a spectrophotometer (NanoDrop 2000, Thermo Fisher Scientific, Shanghai, China). cDNA was synthesized with 1 µg total RNA using a reverse-transcription PCR kit (Takara, Dalian, China). Real-time quantitative PCR was performed with Power SYBR Green RT-PCR reagents (Biorad, Shanghai, China) using the QuantStudioTM 6 Flex System (Applied Biosystems, USA). The PCR conditions were 1 cycle of 95°C for 30 s and 40 cycles of 95°C for 15 s followed by 60°C for 1 min. Data were analysed by 2-delta CT method with *Bactin* as the reference gene. Sequences of the primers were given in Supplementary Table S1.

### Western blot analysis

Western blot analysis was performed as previously reported [25]. Briefly, total proteins were extracted from 3T3-L1 adipocytes and adipose tissues using cell lysis buffer (Beyotime, Shanghai, China) supplemented with protease inhibitor (Roche, Mannheim, Germany). Twenty-microgram protein of each sample was separated in 10% polyacrylamide gel (Beyotime, Shanghai, China) and then was transferred onto PVDF membrane (BioRad). The membranes were blocked in 1 × TBS solution containing 1% bovine serum albumin (Beyotime) and 0.05% Tween-20 (BioRad, Shanghai, China) for 1 h at room temperature, followed by incubation with the appropriate primary antibodies overnight at 4°C. Antibodies to ACC1 (1:2000), pERK (1:2000), ERK (1:2000), pNFκB (1:2000), NFκB (1:2000), Caspase 3 (1:2000) and Tubulin (1:10,000) were obtained from Cell Signalling Technology (Shanghai, China). The membranes were then incubated with their appropriate second antibodies (Cell Signalling Technology) for 1 h after 6 times washing with 1 × TBST solution. After another 6 time-wash in 1 × TBST solution, the protein signalling on the membranes was obtained using the ChemiDocTM XRS+ imaging system (BioRad) with ECL western blotting detection reagent (BioRad). The blots were quantified by Image Lab 5.1 Software (BioRad).

### Statistical analysis

Data were analysed using the software of SAS 9.4. One-way ANOVA with post hoc analysis was applied for multiple comparisons; independent t-test was utilized to compare the difference between two groups; repeated-measures ANOVA was used to analyse the statistical difference of GTT and ITT study. All data were presented as mean ± SEM and *P* value <0.05 was considered statistically significant.

## Results

### *UA induced the expression of* Il6 *and chemokine genes in 3T3-L1 adipocytes*

**Figure 1.**
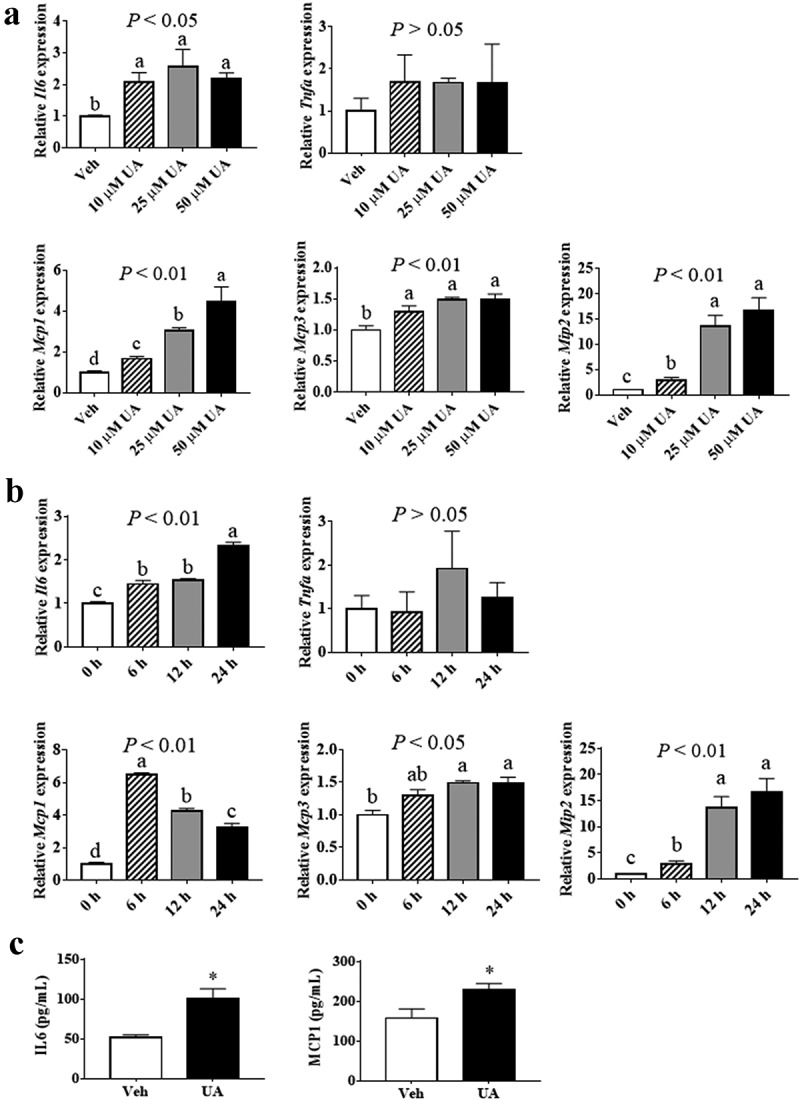
UA induced the expression of *Il6* and chemokine genes in cultured adipocytes. (a) 3T3-L1 adipocytes were treated with vehicle, 10 µM, 25 µM or 50 µM UA for 12 h. Gene expression levels of *Il6, Tnfa, Mcp1, Mcp3* and *Mip2* in the cells were analysed. (b) 3T3-L1 adipocytes were treated with vehicle or 25 µM UA for the indicated time. Gene expression levels of *Il6, Tnfa, Mcp1, Mcp3* and *Mip2* in the cells were detected. (c) 3T3-L1 adipocytes were treated with vehicle or 25 µM UA for 12 h, concentrations of IL6 and MCP1 in the cell culture medium were measured. N = 3 per group. *P* values in the bar graph represent the results of one-way ANOVA analysis. Different letters of a, b and c on the bars indicate significant difference among the groups. Results represented one of three independently performed experiments

Results showed that the expression of *Il6*, but not *Tnfa*, could be induced by UA in adipocytes as compared with control group (Figure 1(a,b)). Meanwhile, the expressions of chemokines *Mcp1, Mcp3* and *Mip2* were increased by UA treatment in adipocytes, in a dose and time dependent manner (Figure 1(a,b)). Concentrations of IL6 and MCP1 in cell culture medium were also increased by UA treatment compared with the control group (Figure 1(c)).

### UA induced the expression of interleukin and chemokine genes in the adipose tissue of DIO mice

**Figure 2.**
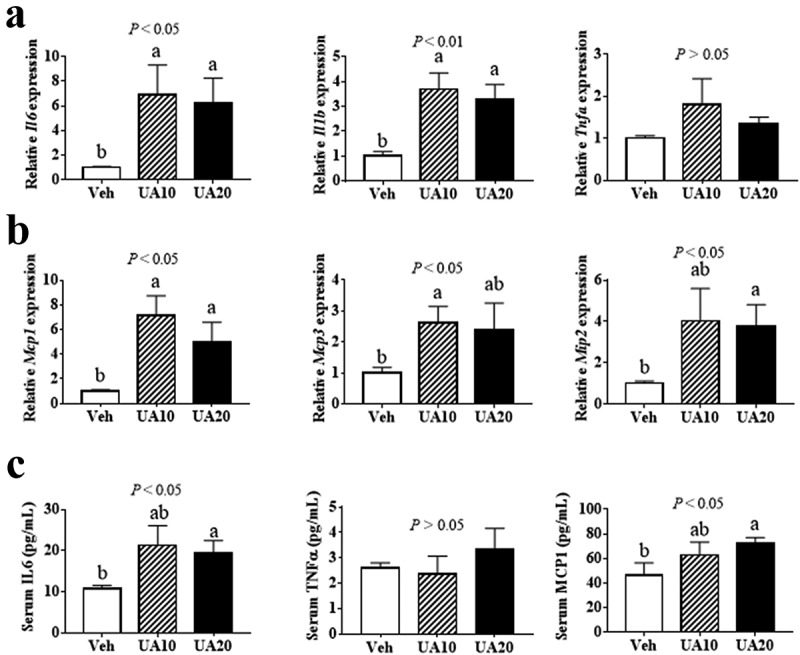
UA promoted the expression of interleukin and chemokine genes in the adipose tissue of DIO mice. DIO mice were treated with UA or vehicle for 3 d. (a) *Il6, Il1b* and *Tnfa* expression levels in gonadal fat tissues. (b) *Mcp1, Mcp3* and *Mip2* expression levels in gonadal fat tissues. (c) Concentrations of IL6, TNFα and MCP1 in mouse serum. N = 5 mice per group. *P* values in the bar graph represent the results of one-way ANOVA analysis. Different letters of a and b on the bars indicate significant difference among the groups

UA has been reported to suppress inflammatory gene expression in BEAS-2B bronchial epithelial cells and spinal cord, while induce inflammation in peritoneal macrophages [14,21,26]. And, our in vitro data had revealed that UA induced the expression of *Il6* and chemokine genes *Mcp1, Mcp3* and *Mip2* in adipocytes. To investigate the in vivo effect of UA on adipose inflammatory and chemokine gene expression, diet-induced obese (DIO) mice were treated with UA for 3 d. Results showed that UA treatment did not change the body weight, blood glucose level or adipose tissue weight (Supplementary Figure S1A-D). The expression of *Il6, Il1b, Mcp1, Mcp3* and *Mip2* in gonadal adipose tissue (gWAT) were remarkably induced by UA, as compared to those in the control group (Figure 2(a,b)). However, UA did not change the expression of *Tnfa* in the gWAT (Figure 2(a)). Further study indicated that serum levels of IL6 and MCP1 were increased by 20 mg/kg UA treatment as compared to those in the control mice (Figure 2(c)). No difference of serum TNFα level was observed between the UA treated mice and control mice (Figure 2(c)).

### UA induced the expression of interleukin and chemokine genes in the adipose tissue of lean mice

**Figure 3.**
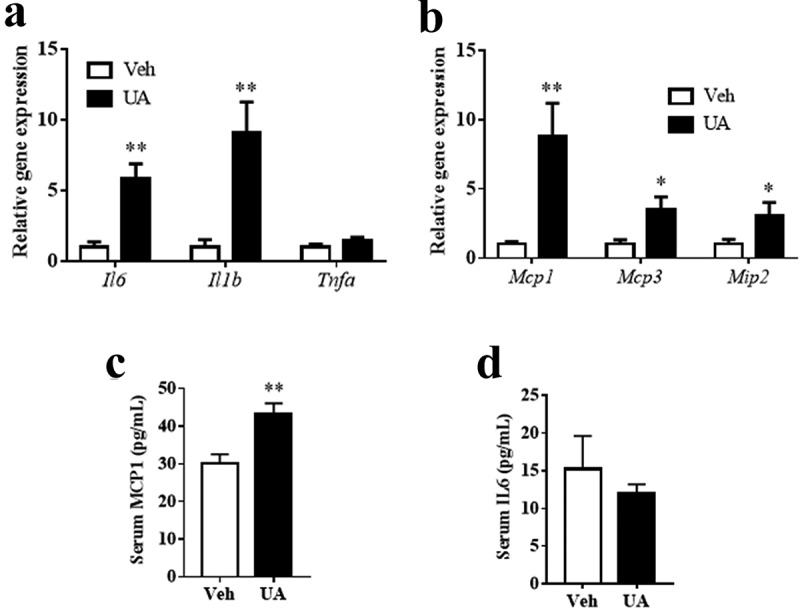
UA induced the expression of interleukin and chemokine genes in the adipose tissue of lean mice. Normal chow fed mice were treated with UA (n = 6) or vehicle (n = 7) for 3 d. (a) Gene expression levels *Il6, Il1b* and *Tnfa* in gonadal fat tissues. (b) *Mcp1, Mcp3* and *Mip2* expression levels in gonadal fat tissues. (c) Serum levels of IL6. (d) Serum levels of MCP1. *P < 0.05, **P < 0.01 Veh VS UA

The obese individuals have higher adipose tissue inflammation level and macrophage content than the lean individuals [6]. Thus, we next tested the effect of UA on adipose tissue inflammation in lean mice. Similar to the results in DIO mice, UA administration did not change the body weight or the adipose tissue weight as compared to the control group (Supplementary Figure S2A-C). Gene expression of *Il6, Il1b, Mcp1, Mcp3* and *Mip2*, but not *Tnfa*, were upregulated by UA in the gWAT of lean mice, as compared with those of the control mice (Figure 3(a,b)). Besides, serum level of MCP1 was significantly higher in the UA treated mice than that in the control mice (Figure 3(c)). However, no significant difference of serum IL6 was observed between the UA group and control group (Figure 3(d)).

### UA inhibited the expression of lipogenic genes in 3T3-L1 adipocytes

**Figure 4.**
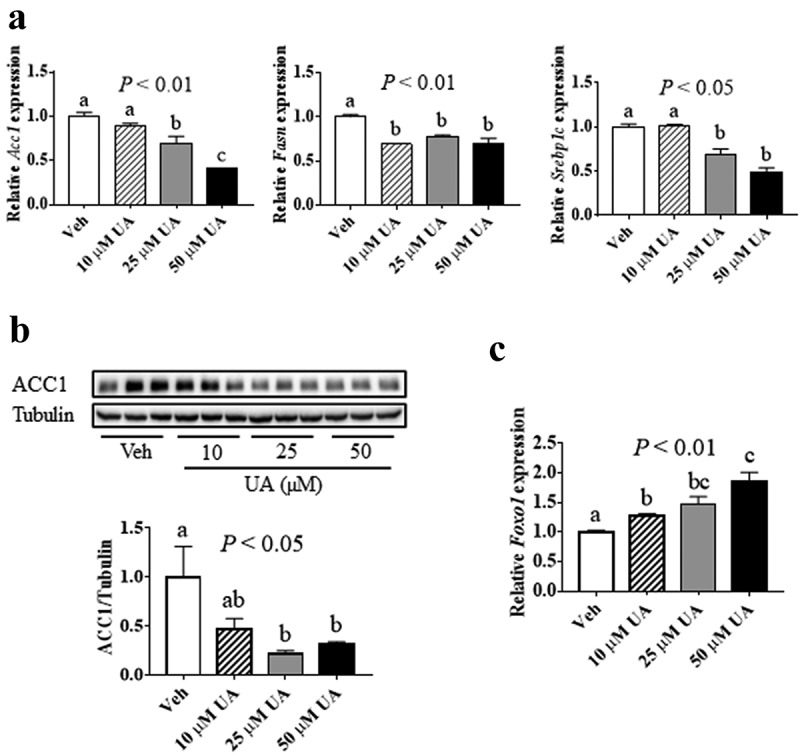
UA suppressed lipogenic gene expression in adipocytes. 3T3-L1 adipocytes were treated with vehicle, 10 µM, 25 µM or 50 µM UA for 12 h. (a) Gene expression levels of *Acc1, Fasn* and *Srebp1c*. (b) ACC1 protein levels in the cells. (c) Gene expression levels of *Foxo1. P* values in the bar graph represent the results of one-way ANOVA analysis. Different letters of a, b and c on the bars indicate significant difference among the groups. N = 3 per group. Results represented one of three independently performed experiments

It has been reported that UA suppressed lipogenesis in liver and inhibited adipogenesis in 3T3-L1 preadipocytes [13]. Here, we tested the effect of UA on lipogenic genes expression in 3T3-L1 mature adipocytes. Results showed that UA significantly suppressed the gene expression of *Acc1, Fasn* and Sterol regulatory element-binding protein 1 c (*Srebp1c*) in adipocytes at the doses of 25 and 50 µM, as compared to the control group (Figure 4(a)). Besides, the protein level of ACC1 was decreased by 25 µM and 50 µM UA, as compared to the control group (Figure 4(b)). As forkhead box protein O1 (FOXO1) has been reported to suppress lipogenesis [27]. We investigated *Foxo1* expression in the adipocytes. Result showed that the *Foxo1* expression was remarkably increased by UA treatment compared with that in the control cells (Figure 4(c)). These data indicated that UA could suppress lipogenesis in cultured adipocytes.

### UA stimulated white adipose tissue to recruit CD14+ monocytes

**Figure 5.**
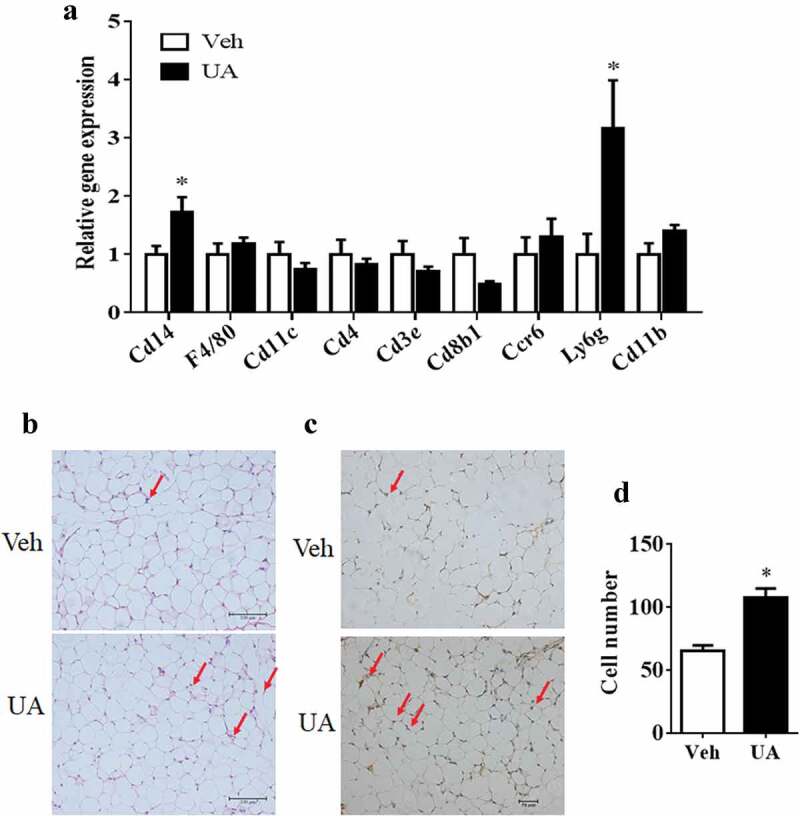
UA stimulated white adipose tissue to recruit CD14+ monocytes. Normal chow fed male mice were treated with UA (n = 6) or vehicle (n = 7) for 3 d. (a) Gene expression levels of *F4/80, Cd11 c, Cd14, Cd4, Cd3e, Cd8b1, Ccr6, Ly6g* and *Cd11b* in gonadal fat tissues. (b) H&E staining images for gonadal fat tissues. Red arrows indicate crown-like structures. (c) Images for CD14 immunohistochemical stain of gonadal fat tissues. Red arrows indicate CD14 positive monocytes. (d) Average numbers of CD14 positive monocytes per images. *P < 0.05 Veh VS UA

Increased expression and secretion of chemokines in adipose tissue could stimulate immune cells to infiltrate into adipose tissue [6,28]. Thus, expression of the marker genes of immune cells was measured in the adipose tissue. Results showed that the expression of *F4/80* and *Cd11 c*, markers of macrophage, were not changed by UA treatment in the gWAT of lean mice, as compared to those in the control mice (Figure 5(a)). For the marker genes of monocytes, primary T cells and neutrophil, UA only increased the expression of *Cd14* and *Ly6g*, but not *Cd4, Cd3e, Cd8b1, Ccr6* or *Cd11b* in the gWAT of lean mice compared with the control group (Figure 5(a)). Similarly, only the expression of *Cd14* and *Ly6g* were increased by UA in the gWAT of DIO mice (Supplementary Figure S3).

H&E staining and immunohistology straining were then performed to check the CD14+ cells in the adipose tissue of lean mice. Results showed that the number of CD14+ monocytes was increased in the adipose tissues of UA treated mice as compared to that in the control mice (Figure 5(b-d)).

### UA administration-impaired mouse glucose tolerance test

**Figure 6.**
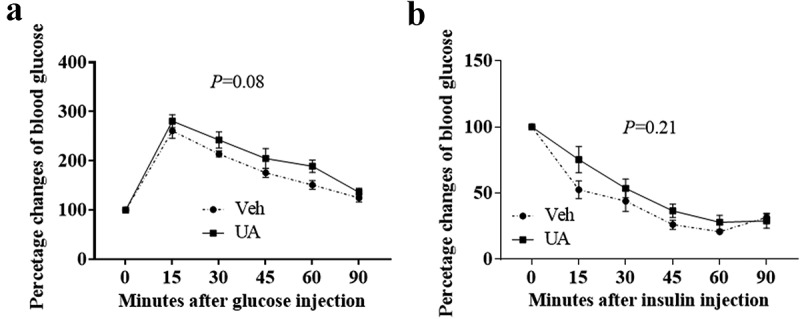
UA administration trended to impair glucose tolerance test in DIO mice. Diet-induced obese mice were daily injected with UA or vehicle for 3 d (n = 7 per group). Glucose tolerance test (a) was performed on day 4, and insulin tolerance test (b) was performed on day 7 of UA treatment. *P* values in the bar graph represent the results of repeated-measures ANOVA analysis

It was reported that chronic adipose tissue inflammation could impair insulin sensitivity and induce lipids metabolic disorder [4]. Thus, we further analysed the glucose tolerance test, insulin tolerance test and lipids metabolic gene expression. Results showed that UA trended to impair mouse glucose tolerance test as compared to the control group (Figure 6(a)). However, insulin tolerance test was not changed by UA treatment (Figure 6(b)). Besides, short-term UA administration did not change the expression of lipogenic or lipolytic genes in lean or DIO mice compared with the control group (Supplementary Figure S4A,B).

### *UA upregulated expression of* Tlr4 *and phosphorylation levels of ERK and NFκB in adipocytes*

**Figure 7.**
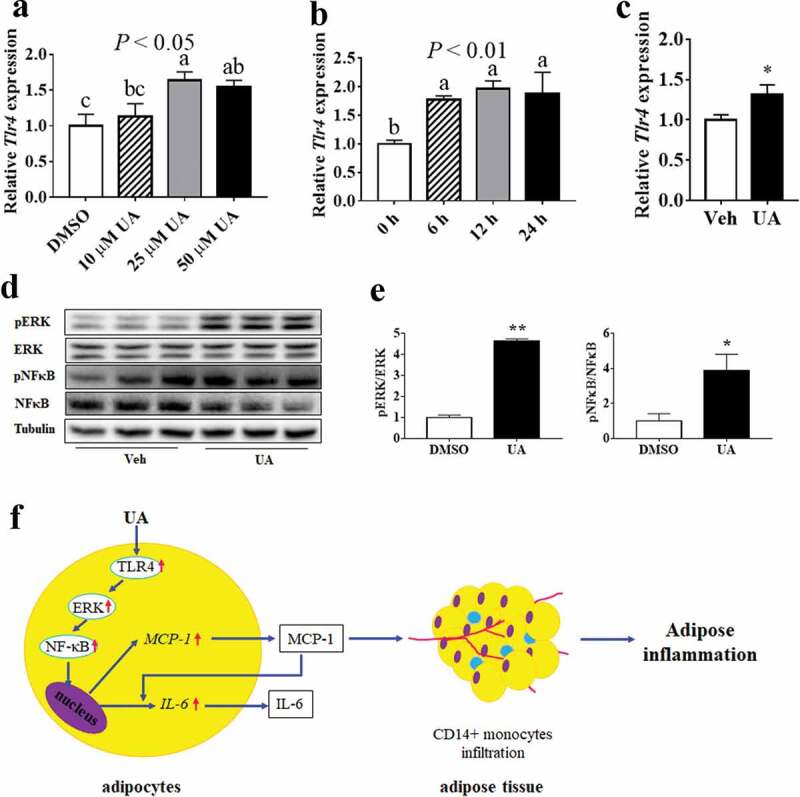
UA upregulated the expression of *Tlr4* and the phosphorylation levels of ERK and NFκB. (a) *Tlr4* expression levels in 3T3-L1 adipocytes which were treated with vehicle, 10 µM, 25 µM or 50 µM UA for 12 h (n = 3 per group). (b) *Tlr4* expression levels in 3T3-L1 adipocytes which were treated with 25 µM UA or vehicle for the indicated time (n = 3 per group). (c) *Tlr4* expression levels in the gonadal adipose tissue of lean mice which were treated with vehicle or UA (n = 6–7 per group). (d,e) Phosphorylation levels of ERK and NFκB in the 3T3-L1 adipocytes which were treated with 25 µM UA or vehicle for 30 min (n = 3 per group). (f) Illustration for UA stimulating adipose tissue to recruit CD14+ monocytes. *P < 0.05, **P < 0.01 Veh VS UA. *P* values in the bar graph represent the results of one-way ANOVA analysis. Different letters of a, b and c on the bars indicate significant difference among the groups. Results for cell studies represented one of three independently performed experiments

As the inflammatory gene expression is regulated by TLR4 in macrophages and epithelial cells, so the expression of *Tlr4* was tested in both cultured adipocytes and adipose tissue. Results showed that UA could induce the expression of *Tlr4* in a dose-dependent manner in 3T3-L1 adipocytes, as compared to the control group (Figure 7(a)). Besides, UA treatment, from 6 h to 24 h, could induce the expression of *Tlr4* (Figure 7(b)). In vivo study also showed that the expression of *Tlr4* was increased by UA in the gWAT of lean mice as compared that of the control mice (Figure 7(c)).

ERK/NFκB pathway was reported to be involved in the regulation of inflammation. Our data showed that the phosphorylation levels of both ERK and NFκB were upregulated by UA in 3T3-L1 adipocytes, as compared to the control group (Figure 7(d,e)). However, the protein level of Caspase 3, another regulator for inflammatory genes, was not changed by UA treatment in adipocytes compared with the control group (Supplementary Figure S5A,B).

## Discussion

Adipose tissue is the major organ for excess energy storage, as well as an endocrine organ which secretes inflammatory factors and chemokines. Here, we found that short-time treatment with UA induced the production of IL6, IL1β, MCP1, MCP3 and MIP2 in both adipocytes and adipose tissue, and subsequently stimulated the adipose tissue to recruit CD14+ monocytes. Besides, we also revealed that short-time UA treatment trended to impair glucose tolerance test in diet-induced obese mice. Our results are important for the functional study of UA.

Adipose tissue secretes many cytokines, such as IL6, TNFα, MCP1 and MIP2, who play important roles in the regulation of metabolism and immunity [1,4,29,30]. Our results suggested that UA remarkably increased the expression of interleukin and chemokine genes, but not *Tnfa*, in both cultured adipocytes and mouse adipose tissue. Meanwhile, serum concentration of MCP1 was induced by UA treatment in both DIO mice and lean mice, while serum IL6 level was increased by UA treatment in lean mice. Previous studies have reported that UA inhibited inflammation in many tissues and cells, including liver, kidney, spinal cord, intestine and macrophages [9,16,18,23,26,31]. However, some other studies found that UA enhanced proinflammatory factors expression in resting macrophages and mouse skin [14,15,20]. Thus, UA has both positive and negative effects on the expression of inflammatory cytokines in different cell types and tissues. Our study demonstrated that UA stimulated the expression of interleukins *Il6* and *Il1b* and chemokines *Mcp1, Mcp3* and *Mip2* in adipocytes and adipose tissue. It is interesting that only the expression of *Il6*, but not *Tnfa*, was elevated by UA in adipocytes. That might be because MCP1 could induce the expression of *Il6*, but not *Tnfa* [32]. It has been reported that IL6 could induce hepatic insulin resistance [33,34]. Thus, short-term UA administration might impair hepatic insulin sensitivity by stimulating adipose tissue to secret IL6. Our data also demonstrated that UA administration trended to impair glucose tolerance test in DIO mice. The effect of long-term UA administration on adipose tissue inflammation and insulin sensitivity needs further study to be explored.

Previous study indicated that adipocyte-secreted MCP1 could stimulate the infiltration of macrophages into adipose tissue via MCP1/CCR2 pathway [28]. Besides, MCP1 could also recruitment T lymphocytes and other immune cells to inflammation sites [35,36]. Our results indicated that UA markedly increased the expression of *Cd14* and the number of CD14 positive monocytes in adipose tissue, while the expression of macrophage marker genes *F4/80* and *Cd11 c* were not changed. These data indicated that short-time UA administration stimulated the white adipose tissue to recruit CD14 positive monocytes, but not macrophages. CD14 has been reported to induced inflammation and insulin resistance in adipose tissue [37,38]. Thus, short-term UA administration might impair adipose tissue insulin sensitivity by recruit CD14+ monocytes. It was reported that activated monocytes could be transferred to macrophages in adipose tissue [39]. The reason why we did not observe the change of macrophage marker in UA treated adipose tissue might be because our study was a short-time study. Maybe, longer time of UA administration could change the content of macrophages in adipose tissue. However, the hypothesis needs further study to be confirmed.

According to previous studies, UA regulated the expression of *TNFα, IL6* and *IL1β* via the key inflammatory transcription factor NFκB in activated T cells, B cells and macrophages [20,31]. Results of Ghosh suggested that TLR4 enhanced *Il6, Tnfa* and *Mcp1* expression through NFκB pathway in adipose tissue of ageing mice [40]. Another study showed that UA decreased *Il6, Tnfa* and *Il1b* expression by inhibiting TLR4/NFκB pathway in mouse macrophages [22]. Besides, Thompson and colleagues suggested that NFκB stimulated the expression of MCP1 in astrocytes [41]. Our results showed that UA stimulated *Tlr4* expression and phosphorylation level of NFκB in adipocytes. Therefore, UA might up-regulate the production of interleukins and chemokines through the TLR4/NFκB signalling pathway in adipocytes (Figure 7(f)).

Suppressed ERK/NFκB signalling was reported to mediate UA inhibiting inflammation in liver [23]. Study of Ma et al. suggested that UA might inhibit ERK/NFκB pathway via inactivation of Caspase 3 [21]. Our results found that though UA activated ERK/NFκB signalling pathway, but it did not change Caspase 3 protein level. These results indicated that UA might also induce the expression of interleukins and chemokines by activating ERK/NF-κB pathway (Figure 7(f)). The protein level of Caspase 3 was not affected by UA in our study might be because we used different cells from other studies. Ma *et al*. used BEAS-2B epithelial cells [21]. Wu *et al*., who found that UA stimulated ERK/NFκB pathway by activating Caspase 3, used human osteosarcoma cells [42]. However, 3T3-L1 adipocytes were used in our study. Besides, FOXO1 was reported to induce the expression of *Il6* and *Mcp1* in adipocytes [43]. The expression of *Foxo1* was increased by UA in our study. Thus, FOXO1 might also be involved in UA inducing the expression of interleukins and chemokines in adipocytes.

Fat deposition is closely related to the expansion of adipose tissue mass. Overload of fat leads to adipose tissue hypertrophy, and even obesity [44]. Thus, discovery of chemicals and components in herbs those can reduce body fat accumulation is very important for human health. As a triterpenoid extracted from plants and herbs, UA has shown its function on suppressing lipogenesis in liver and on inhibiting adipogenesis in 3T3-L1 preadipocytes [10–12,45]. Similarly, our study demonstrated that UA inhibited lipogenic gene expression in cultured adipocytes.

Many studies have reported that UA decreased adipose accumulation in DIO mice and rats [12,13,46–48]. However, in those studies, the direct targets of UA seemed not to be the adipose tissue itself, but the liver and muscle. Because these reports did not show the expression of lipogenic genes or lipolytic genes in the adipose tissue. We observed neither the change of adipose tissue weight nor the change of lipogenic gene expression by UA treatment, in DIO mice or lean mice. The different results on adipose tissue weight change between our study and others might be because our study was a short-time trail with only 3-d treatment, while previous studies treated animals for at least 5 weeks. The study of Sundaresan et al. indicated that 5 mg/kg BW UA administration for 5 weeks caused a significant reduction in epididymal fat tissue weight [48]. Jia and colleagues suggested that only 200 but not 50 mg/kg BW UA treatment for 8 weeks could reduce adipose accumulation in DIO mice [13]. One study suggested that DIO mice took feed with 0.14% UA (w/w) for 6 weeks had lower epididymal fat weight than the control mice [46], whereas Li et al. showed that intake of feed supplemented with 0.25% UA (w/w), but not the feed with 0.125% UA, for 6 weeks could decrease fat content in DIO rats [12]. Another study showed that DIO mice, who drank water containing 0.05% UA (w/v) (equivalent to a dose of 10 mg/kg of body weight based on water consumption), accumulated less visceral fat after 15-week treatment than the control mice [47]. These studies indicate that UA has the potent to suppress fat accumulation. However, the treatment conditions and the molecular mechanisms still need to be defined.

FOXO1 has been reported to suppress the expression of *SREBP1c* [27], a transcriptional factor which upregulates *ACC1* and *FASN* expression [49,50]. Our study demonstrated that UA stimulated *Foxo1* expression and decreased *Srebp1c* expression in adipocytes. Thus, UA might suppress lipid synthesis through FOXO1/SREBP1c pathway in adipocytes. Besides, IL6 was reported to inhibit *Fasn* expression in DIO mouse liver [51]. Our results showed that *Il6* expression was significantly upgraded by UA in adipocytes. Therefore, UA might also suppress lipids synthesis through IL6 pathway in adipocytes. However, the exact mechanisms need further studies to be elucidated.

In summary, our study indicated that short-term UA administration could stimulate adipose tissue to recruit CD14+ monocytes by inducing the adipocytes to produce interleukins and chemokines via the TLR4/NFκB pathway.

## Supplementary Material

Supplemental Material

## References

[1] Ahima RS. Adipose tissue as an endocrine organ. Obesity (Silver Spring). 2006;14(Suppl 5):242S–249S. 17021375 10.1038/oby.2006.317

[2] Feng B, Jiao P, Nie Y, et al. Clodronate liposomes improve metabolic profile and reduce visceral adipose macrophage content in diet-induced obese mice. PLoS One. 2011;6:e24358. 21931688 10.1371/journal.pone.0024358 PMC3171445

[3] Feng B, Zhang T, Xu H. Human adipose dynamics and metabolic health. Ann N Y Acad Sci. 2013;1281:160–177. 23317303 10.1111/nyas.12009 PMC3618577

[4] Reilly SM, Saltiel AR. Adapting to obesity with adipose tissue inflammation. Nat Rev Endocrinol. 2017;13:633–643. 28799554 10.1038/nrendo.2017.90

[5] Scherer T, O’Hare J, Diggs-Andrews K, et al. Brain insulin controls adipose tissue lipolysis and lipogenesis. Cell Metab. 2011;13:183–194. 21284985 10.1016/j.cmet.2011.01.008 PMC3061443

[6] Xu H, Barnes GT, Yang Q, et al. Chronic inflammation in fat plays a crucial role in the development of obesity-related insulin resistance. J Clin Invest. 2003;112:1821–1830. 14679177 10.1172/JCI19451 PMC296998

[7] Guilherme A, Virbasius JV, Puri V, et al. Adipocyte dysfunctions linking obesity to insulin resistance and type 2 diabetes. Nat Rev Mol Cell Biol. 2008;9:367–377. 18401346 10.1038/nrm2391 PMC2886982

[8] Kahn CR, Wang G, Lee KY. Altered adipose tissue and adipocyte function in the pathogenesis of metabolic syndrome. J Clin Invest. 2019;129:3990–4000. 31573548 10.1172/JCI129187 PMC6763230

[9] Ikeda Y, Murakami A, Ohigashi H. Ursolic acid: an anti- and pro-inflammatory triterpenoid. Mol Nutr Food Res. 2008;52:26–42. 18203131 10.1002/mnfr.200700389

[10] Li Y, Kang Z, Li S, et al. Ursolic acid stimulates lipolysis in primary-cultured rat adipocytes. Mol Nutr Food Res. 2010;54:1609–1617. 20521271 10.1002/mnfr.200900564

[11] He Y, Li Y, Zhao T, et al. Ursolic acid inhibits adipogenesis in 3T3-L1 adipocytes through LKB1/AMPK pathway. PLoS One. 2013;8:e70135. 23922935 10.1371/journal.pone.0070135 PMC3724828

[12] Li S, Liao X, Meng F, et al. Therapeutic role of ursolic acid on ameliorating hepatic steatosis and improving metabolic disorders in high-fat diet-induced non-alcoholic fatty liver disease rats. PLoS One. 2014;9:e86724. 24489777 10.1371/journal.pone.0086724 PMC3906058

[13] Jia Y, Kim S, Kim J, et al. Ursolic acid improves lipid and glucose metabolism in high-fat-fed C57BL/6J mice by activating peroxisome proliferator-activated receptor alpha and hepatic autophagy. Mol Nutr Food Res. 2015;59:344–354. 25418615 10.1002/mnfr.201400399

[14] Ikeda Y, Murakami A, Fujimura Y, et al. Aggregated ursolic acid, a natural triterpenoid, induces IL-1beta release from murine peritoneal macrophages: role of CD36. J Immunol. 2007;178:4854–4864. 17404266 10.4049/jimmunol.178.8.4854

[15] Ikeda Y, Murakami A, Nishizawa T, et al. Ursolic acid enhances cyclooxygenases and tumor necrosis factor-alpha expression in mouse skin. Biosci Biotechnol Biochem. 2006;70:1033–1037. 16636478 10.1271/bbb.70.1033

[16] Chun J, Lee C, Hwang SW, et al. Ursolic acid inhibits nuclear factor-kappaB signaling in intestinal epithelial cells and macrophages, and attenuates experimental colitis in mice. Life Sci. 2014;110:23–34. 24992474 10.1016/j.lfs.2014.06.018

[17] Kim SH, Hong JH, Lee YC. Ursolic acid, a potential PPARgamma agonist, suppresses ovalbumin-induced airway inflammation and Penh by down-regulating IL-5, IL-13, and IL-17 in a mouse model of allergic asthma. Eur J Pharmacol. 2013;701:131–143. 23201068 10.1016/j.ejphar.2012.11.033

[18] Li J, Li N, Yan S, et al. Ursolic acid alleviates inflammation and against diabetes induced nephropathy through TLR4mediated inflammatory pathway. Mol Med Rep. 2018;18:4675–4681. 30221655 10.3892/mmr.2018.9429

[19] Zhang T, Su J, Guo B, et al. Ursolic acid alleviates early brain injury after experimental subarachnoid hemorrhage by suppressing TLR4-mediated inflammatory pathway. Int Immunopharmacol. 2014;23:585–591. 25466266 10.1016/j.intimp.2014.10.009

[20] You HJ, Choi CY, Kim JY, et al. Ursolic acid enhances nitric oxide and tumor necrosis factor-alpha production via nuclear factor-kappaB activation in the resting macrophages. FEBS Lett. 2001;509:156–160. 11741581 10.1016/s0014-5793(01)03161-1

[21] Ma X, Zhang Y, Wang Z, et al. Ursolic acid, a natural nutraceutical agent, targets Caspase3 and alleviates inflammation-associated downstream signal transduction. Mol Nutr Food Res. 2017;61:1700332. 10.1002/mnfr.201700332 PMC5765441 28801966

[22] Jang SE, Jeong JJ, Hyam SR, et al. Ursolic acid isolated from the seed of Cornus officinalis ameliorates colitis in mice by inhibiting the binding of lipopolysaccharide to Toll-like receptor 4 on macrophages. J Agric Food Chem. 2014;62:9711–9721. 25213465 10.1021/jf501487v

[23] Ma JQ, Ding J, Zhang L, et al. Ursolic acid protects mouse liver against CCl4-induced oxidative stress and inflammation by the MAPK/NF-kappaB pathway. Environ Toxicol Pharmacol. 2014;37:975–983. 24727148 10.1016/j.etap.2014.03.011

[24] Huang X, Jiang D, Zhu Y, et al. Chronic high dose zinc supplementation induces visceral adipose tissue hypertrophy without altering body weight in mice. Nutrients. 2017;9:E1138. 29057818 10.3390/nu9101138 PMC5691754

[25] Feng B, Huang X, Jiang D, et al. Endoplasmic reticulum stress inducer tunicamycin alters hepatic energy homeostasis in mice. Int J Mol Sci. 2017;18:E1710. 28777337 10.3390/ijms18081710 PMC5578100

[26] Zhang Y, Song C, Li H, et al. Ursolic acid prevents augmented peripheral inflammation and inflammatory hyperalgesia in high-fat diet-induced obese rats by restoring downregulated spinal PPARalpha. Mol Med Rep. 2016;13:5309–5316. 27108888 10.3892/mmr.2016.5172

[27] Deng X, Zhang W, I OS, et al. FoxO1 inhibits sterol regulatory element-binding protein-1c (SREBP-1c) gene expression via transcription factors Sp1 and SREBP-1c. J Biol Chem. 2012;287:20132–20143. 22511764 10.1074/jbc.M112.347211 PMC3370196

[28] Kanda H, Tateya S, Tamori Y, et al. contributes to macrophage infiltration into adipose tissue, insulin resistance, and hepatic steatosis in obesity. J Clin Invest. 2006;116:1494–1505. 16691291 10.1172/JCI26498 PMC1459069

[29] Vieira-Potter VJ. Inflammation and macrophage modulation in adipose tissues. Cell Microbiol. 2014;16:1484–1492. 25073615 10.1111/cmi.12336

[30] Grant RW, Dixit VD. Adipose tissue as an immunological organ. Obesity (Silver Spring). 2015;23:512–518. 25612251 10.1002/oby.21003 PMC4340740

[31] Checker R, Sandur SK, Sharma D, et al. Potent anti-inflammatory activity of ursolic acid, a triterpenoid antioxidant, is mediated through suppression of NF-kappaB, AP-1 and NF-AT. PLoS One. 2012;7:e31318. 22363615 10.1371/journal.pone.0031318 PMC3282718

[32] Ziraldo C, Vodovotz Y, Namas RA, et al. Central role for MCP-1/CCL2 in injury-induced inflammation revealed by in vitro, in silico, and clinical studies. PLoS One. 2013;8:e79804. 24312451 10.1371/journal.pone.0079804 PMC3849193

[33] Matsubara T, Mita A, Minami K, et al. PGRN is a Key Adipokine Mediating High Fat Diet-Induced Insulin Resistance and Obesity through IL-6 in Adipose Tissue. Cell Metab. 2012;15:38–50. 22225875 10.1016/j.cmet.2011.12.002

[34] Kim JH, Kim J, Liu H-Y, et al. Regulation of IL-6 induced hepatic insulin resistance by mtor through the STAT3-SOCS3 pathway. J Biol Chem. 283:708–715. 10.1074/jbc.M708568200 17993646

[35] Karimian MS, Pirro M, Majeed M, et al. Curcumin as a natural regulator of monocyte chemoattractant protein-1. Cytokine Growth Factor Rev. 2017;33:55–63. 27743775 10.1016/j.cytogfr.2016.10.001

[36] Hayashida K, Nanki T, Girschick H, et al. Synovial stromal cells from rheumatoid arthritis patients attract monocytes by producing MCP-1 and IL-8. Arthritis Res. 2001;3:118–126. 11178119 10.1186/ar149 PMC17828

[37] Fernández-Real JM, Pérez Del Pulgar S, Luche E, et al. CD14 modulates inflammation-driven insulin resistance. Diabetes. 2011;60:2179–2186. 21700881 10.2337/db10-1210 PMC3142089

[38] Luche E, Cousin B, Garidou L, et al. Metabolic endotoxemia directly increases the proliferation of adipocyte precursors at the onset of metabolic diseases through a CD14-dependent mechanism. Mol Metab. 2013;2:281–291. 24049740 10.1016/j.molmet.2013.06.005 PMC3773833

[39] Russo L, Lumeng CN. Properties and functions of adipose tissue macrophages in obesity. Immunology. 2018;155:407–417. 30229891 10.1111/imm.13002 PMC6230999

[40] Ghosh AK, O’Brien M, Mau T, et al. Toll-like receptor 4 (TLR4) deficient mice are protected from adipose tissue inflammation in aging. Aging (Albany NY). 2017;9:1971–1982. 28898202 10.18632/aging.101288 PMC5636669

[41] Thompson WL, Van Eldik LJ. Inflammatory cytokines stimulate the chemokines CCL2/MCP-1 and CCL7/MCP-3 through NFkB and MAPK dependent pathways in rat astrocytes. Brain Res. 2009;1287:47–57. 19577550 10.1016/j.brainres.2009.06.081 PMC2725204

[42] Wu CC, Cheng CH, Lee YH, et al. Ursolic acid triggers apoptosis in human osteosarcoma cells via caspase activation and the ERK1/2 MAPK pathway. J Agric Food Chem. 2016;64:4220–4226. 27171502 10.1021/acs.jafc.6b00542

[43] Ito Y, Daitoku H, Foxo FA. increases pro-inflammatory gene expression by inducing C/EBPbeta in TNF-alpha-treated adipocytes. Biochem Biophys Res Commun. 2009;378:290–295. 19026986 10.1016/j.bbrc.2008.11.043

[44] Lafontan M. Adipose tissue and adipocyte dysregulation. Diabetes Metab. 2014;40:16–28. 24139247 10.1016/j.diabet.2013.08.002

[45] Sundaresan A, Radhiga T, Pugalendi KV. Effect of ursolic acid and Rosiglitazone combination on hepatic lipid accumulation in high fat diet-fed C57BL/6J mice. Eur J Pharmacol. 2014;741:297–303. 25149666 10.1016/j.ejphar.2014.07.032

[46] Kunkel SD, Elmore CJ, Bongers KS, et al. Ursolic acid increases skeletal muscle and brown fat and decreases diet-induced obesity, glucose intolerance and fatty liver disease. PLoS One. 2012;7:e39332. 22745735 10.1371/journal.pone.0039332 PMC3379974

[47] Rao VS, de Melo CL, Queiroz MG, et al. Ursolic acid, a pentacyclic triterpene from Sambucus australis, prevents abdominal adiposity in mice fed a high-fat diet. J Med Food. 2011;14:1375–1382. 21612453 10.1089/jmf.2010.0267

[48] Sundaresan A, Harini R, Pugalendi KV. Ursolic acid and rosiglitazone combination alleviates metabolic syndrome in high fat diet fed C57BL/6J mice. Gen Physiol Biophys. 2012;31:323–333. 23047945 10.4149/gpb_2012_037

[49] Kim JB, Sarraf P, Wright M, et al. Nutritional and insulin regulation of fatty acid synthetase and leptin gene expression through ADD1/SREBP1. J Clin Invest. 1998;101:1–9. 9421459 10.1172/JCI1411 PMC508533

[50] Hagiwara A, Cornu M, Cybulski N, et al. Hepatic mTORC2 activates glycolysis and lipogenesis through Akt, glucokinase, and SREBP1c. Cell Metab. 2012;15:725–738. 22521878 10.1016/j.cmet.2012.03.015

[51] Ma Y, Gao M, Sun H, et al. Interleukin-6 gene transfer reverses body weight gain and fatty liver in obese mice. Biochim Biophys Acta. 2015;1852:1001–1011. 25660446 10.1016/j.bbadis.2015.01.017

