# Supplementary material for: Ursolic acid induces the production of IL6 and chemokines in both adipocytes and adipose tissue
Source: Adipocyte. 2020 Sep 2;9(1):523–34. doi: 10.1080/21623945.2020.1814545 (PMC7714451; doi:10.1080/21623945.2020.1814545)
Supplement: Supplemental Material [file KADI_A_1814545_SM6540.docx]

**Ursolic acid induces the production of IL6 and chemokines in both adipocytes and adipose tissue**

**Supplementary Materials**

**Table S1. Primer sequences for real-time PCR**

| gene name | Access number | Forward (5’>3’) | Reverse (5’>3’) |
| --- | --- | --- | --- |
| *Acc1* | NM_133360 | CGGACCTTTGAAGATTTTGTCAGG | GCTTTATTCTGCTGGGTGAACTCTC |
| *Atgl* | AY894805 | CTGTGTGGAACCAAAGGACCTG | GCTACCCGTCTGCTCTTTCATC |
| *B-actin* | NM_007393 | GGCTGTATTCCCCTCCATCG | CCAGTTGGTAACAATGCCATGT |
| *Ccr6* | NM_001190338 | CCTGGGCAACATTATGGTGGT | CAGAACGGTAGGGTGAGGACA |
| *Cd11b* | NM_001082960 | CTTCCAGGGCAGGAGTCGTA | GATGAGAGCCAAGAGCACCAG |
| *Cd11c* | NM_021334 | CTGGATAGCCTTTCTTCTGCTG | GCACACTGTGTCCGAACTCA |
| *Cd14* | NM_009841 | CTCTGTCCTTAAAGCGGCTTAC | GTTGCGGAGGTTCAAGATGTT |
| *Cd3e* | NM_007648 | ATGCGGTGGAACACTTTCTGG | GCACGTCAACTCTACACTGGT |
| *Cd4* | NM_013488 | AGGTGATGGGACCTACCTCTC | GGGGCCACCACTTGAACTAC |
| *Cd8b1* | NM_009858 | CTCTGGCTGGTCTTCAGTATGA | TCTTTGCCGTATGGTTGGTTT |
| *Fasn* | NM_007988 | GGCTCTATGGATTACCCAAGC | CCAGTGTTCGTTCCTCGGA |
| *Foxo1* | NM_019739 | ATGCTCAATCCAGAGGGAGG | ACTCGCAGGCCACTTAGAAAA |
| *F4/80* | NM_010130 | TGACTCACCTTGTGGTCCTAA | CTTCCCAGAATCCAGTCTTTCC |
| *Hsl* | U08188 | TGAAGCCAAAGATGAAGTGAGAC | CTTGACTATGGGTGACGTGTAGAG |
| *Il1b* | NM_008361 | GAAATGCCACCTTTTGACAGTG | TGGATGCTCTCATCAGGACAG |
| *Il6* | NM_031168 | TAGTCCTTCCTACCCCAATTTCC | TTGGTCCTTAGCCACTCCTTC |
| *Srebp1c* | NM_001313979 | AACTGCCCATCCACCGACTC | ATTGATAGAAGACCGGTAGCGC |
| *Ly6g* | NM_001310438 | GACTTCCTGCAACACAACTACC | ACAGCATTACCAGTGATCTCAGT |
| *Mcp1* | NM_011333 | TTAAAAACCTGGATCGGAACCAA | GCATTAGCTTCAGATTTACGGGT |
| *Mcp3* | NM_013654 | AGAAACAAAAGATCCCCAAGAGG | CCAGGGACACCGACTACTG |
| *Mip2* | NM_009140 | CCCAGACAGAAGTCATAGCCA | CTCCTTTCCAGGTCAGTTAGC |
| *Tlr4* | NM_021297 | GCCTTTCAGGGAATTAAGCTCC | GATCAACCGATGGACGTGTAAA |
| *Tnfa* | NM_013693 | GACCCTCACACTCAGATCATCTTCT | CCACTTGGTGGTTTGCTACGA |



**Figure S1. UA treatment did not change the body weight or adipose tissue weight in DIO mice.** DIO mice were treated with UA or vehicle for 3 days. Body weight (**A**), blood glucose levels (**B**), gonadal fat weight (**C**) and white fat weight (**D**) were measured at harvesting. N=5 per group





**Figure S2. UA has no effect on the body weight or white adipose tissue weight in lean mice.** Normal chow fed mice were treated with UA (n=6) or vehicle (n=7) for 3 days. Body weight (**A**), gonadal fat weight (**B**) and white fat weight (**C**) were measured at harvesting.





**Figure S3. UA induced the expression of *Cd14* and *Ly6g* in gWAT of DIO mice.** DIO mice were treated with UA or vehicle for 3 days. Expression of *F4/80*, *Cd11c*, *Cd14*, *Cd4*, *Cd3e*, *Cd8b1*, *Ccr6*, *Ly6g* and *Cd11b* were detected in the gonadal fat tissue. N=5 per group. *P* values in the bar graph represent the results of one-way ANOVA analysis. Different letters of a and b on the bars indicate significant difference among the groups.


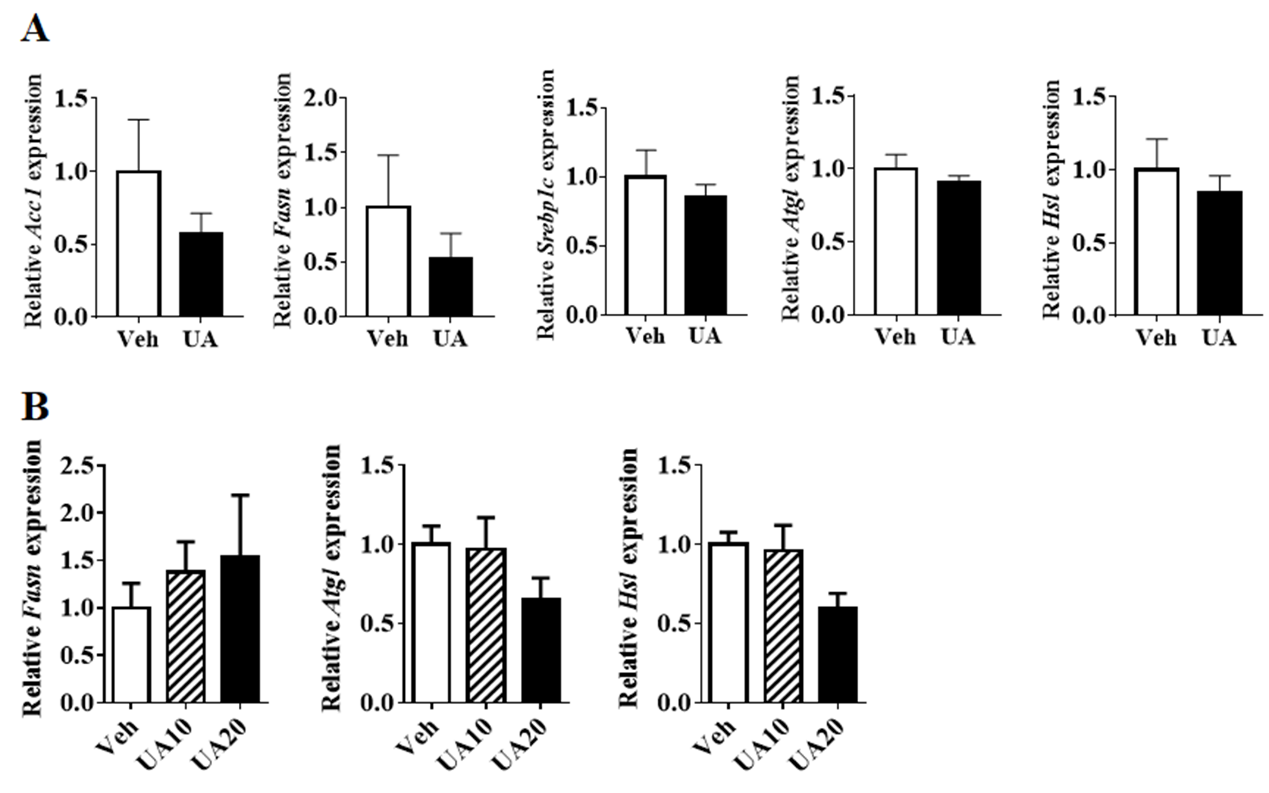


**Figure S4. UA treatment did not change the expression of lipogenic or lipolytic genes in gWAT. (A)** Normal chow fed mice were treated with UA (n=6) or vehicle (n=7) for 3 days. Gene expression of *Acc1*, *Fasn*, *Srebp1c*, *Atgl* and *Hsl* were detected in gonadal fat tissue. **(B)** DIO mice were treated with UA or vehicle for 3 days (N=5 per group). Gene expression of *Fasn*, *Atgl* and *Hsl* were detected in the gonadal fat tissues.


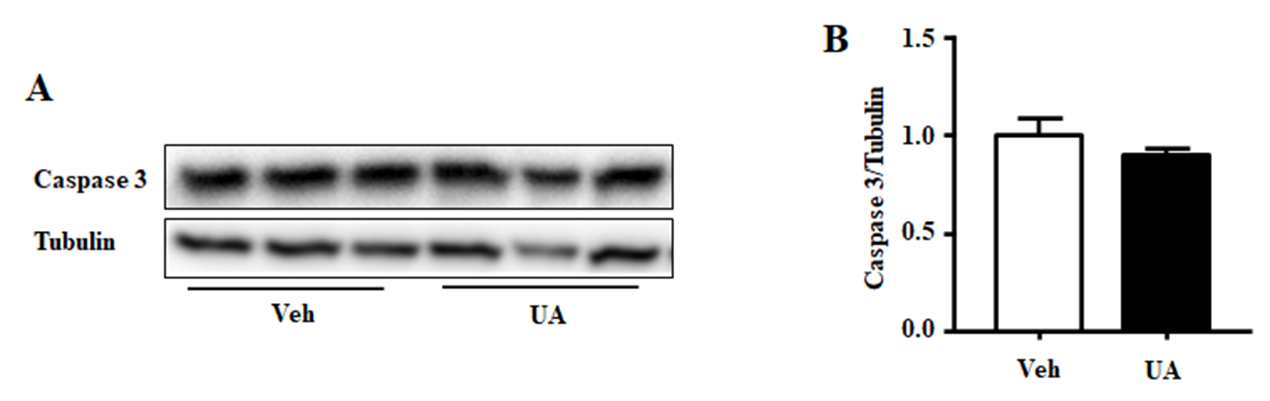


**Figure S5.** **UA treatment did not alter Caspase 3 protein level in 3T3-L1 adipocytes.** 3T3-L1 adipocytes were treated with 25 µM UA or vehicle for 6 h. (**A**) Protein levels of Caspase 3 in the cells. (**B**) Quantification of the blots in A. N=3 per group. Results represented one of three independently performed experiments.
